# Investigating the relationship between anxiety, depression, and forearm muscle deformation in amateur athletes using time-series analysis

**DOI:** 10.3389/fspor.2025.1561808

**Published:** 2025-09-02

**Authors:** Satoshi Shimabukuro, Tamon Miyake, Tomo Akamine, Chihiro Ookubo, Dai Yanagihara, Emi Tamaki

**Affiliations:** ^1^ Department of Engineering, University of the Ryukyus, Nishihara, Okinawa, Japan; ^2^ Department of Physical Therapy, Okinawa Rehabilitation Welfare College, Yonabaru, Okinawa, Japan; ^3^ H2L Inc., Tokyo, Japan; ^4^ Future Robotics Organization, Waseda University, Tokyo, Japan; ^5^ Department of Life Sciences, Graduate School of Arts and Sciences, The University of Tokyo, Tokyo, Japan; ^6^ Department of Systems Innovation, School of Engineering, The University of Tokyo, Tokyo, Japan

**Keywords:** muscle deformation, sports condition, anxiety and depression, autocorrelation coefficient, amateur athlete

## Abstract

**Introduction:**

Conditioning involves the regulation of psychological health, physical fitness, and overall well-being, all of which are essential for optimal athletic performance. While psychological aspects have been extensively studied, the relationship between muscle activity and psychological factors such as anxiety and depression remains underexplored.

**Methods:**

This study investigated the relationship between anxiety, depression, and forearm muscle activity in amateur athletes (mean age: 28 ± 9 years; sports experience: 13 ± 5 years) who participate in sports requiring upper extremity use. Muscle activity was evaluated using autocorrelation coefficients derived from muscle deformation data collected via a sensor array. Anxiety and depression levels were assessed using validated questionnaires, and their associations with muscle deformation were analyzed.

**Results:**

Muscle deformation suggested a task-dependent relationship with psychological factors. A significant correlation was observed between anxiety and Hand tasks (r = −0.57, *p* = 0.004, pfdr = 0.05) and a trend was found between depression and random tasks such as HPI randomness (r = 0.46, *p* = 0.04, pfdr = 0.15).

**Discussion:**

These findings suggest that chronic psychological stress may impair sustained muscle contraction during cyclic movements and promote fixed motor patterns during random tasks. This study elucidates how anxiety and depression affect muscle activity under different task conditions, providing a basis for optimizing training protocols and psychological stress management strategies in athletic conditioning.

## 1 Introduction

Sports science focuses extensively on enhancing athletes’ performance, particularly the critical role of conditioning in improving physical and mental health (1). Conditioning involves optimizing psychological well-being, physical fitness, and overall health, which are essential for peak performance (2, 3). Despite comprehensive efforts, achieving this ideal state presents challenges, including slumps (4), and performance plateaus. Athletes may also face specific issues like “yips” (5, 6)—a sudden loss of fine motor skills and dystonia (7, 8), which stem from psychological stress, physical health concerns, environmental factors, and learning dynamics.

Psychological stress can be divided into acute and chronic stress. Acute stress is a temporary response to external stimuli or stressors. Chronic stress persists for weeks to several months or longer and can affect the body, mind, and behavior (9). Prolonged psychological load disrupts leading to sustained activation of the hypothalamic—pituitary—adrenal (HPA) axis and chronically elevated glucocorticoid secretion (10), Such HPA-axis dysregulation has been implicated in insomnia, depressive symptoms, and the development of stress-related lifestyle disorders (11). Chronic exposure to high levels of glucocorticoids in adulthood has been associated with depressive disorders (12) Additionally, high doses of glucocorticoids are believed to cause muscle dysfunction, including decreased skeletal muscle size, mass, and weakness (10, 13). A chronic stress history reduces basal activity below control levels and abolishes the activation response to acute stress (14).

The reduction of acute activation has been reported to be similar to depression (15). The importance of anxiety and other emotional and personality factors in athletic competition has long been recognized (15–17); performance and competition anxiety in sports are closely related to each other and have been shown to reinforce each other negativelys (18). In addition, Chronic stress changes have also been reported in relation to physical functioning, with low grip strength being cross-sectionally and longitudinally associated with depressive symptoms. (19, 20). However, it is unclear whether anxiety and depression affect physical function, particularly the skeletal muscle status, in athletes. Therefore, this study aimed to determine the relationship between the effects of anxiety and depression on forearm muscle activity in athletes performing various exercise tasks. The findings of this study are expected to provide basic data for stress assessment and for designing targeted mental-intervention strategies in athletic settings.

## 2 Methods

### 2.1 Participations

Twenty-one healthy males (age: 28 ± 9 years, height: 173 ± 7 cm, weight: 70 ± 12 kg, sports experience: 13 ± 5 years) participated in the study (Table 1). The participants were amateur athletes between the ages of 18 and 59 years with at least 5 years of continuous experience in sports involving the upper extremities. Open recruitment was conducted among those who played upper-limb sports such as baseball and golf, in which slumps and yips are known to be common, had at least 5 years of continuous experience, and subjectively experienced a decline in performance within 3 months. Exclusion criteria included cardiac disease (use of a pacemaker), psychiatric disease, no serious illness, and any injury or disease for which treatment or rehabilitation had not been completed. Pregnant or possibly pregnant, have skin atopy, or have a job or hobby (playing a musical instrument) that involves wrist use (Table 2).

**Table 1:** Participants in this study.

No.	Age (years old)	Height (cm)	Weight (kg)	Sports	Experience (years)
1	48	166	79.5	Golf	8
2	45	172	63.9	Golf	15
3	36	179	71.2	Golf	8
4	50	182	90.2	Golf	20
5	20	170	52.1	Badminton	6
6	26	175	66.3	Tennis	11
7	19	165	57.3	Tennis	7
8	22	180	67.7	Tennis	10
9	23	167	72.6	Tennis	14
10	24	168	52.3	Tennis	14
11	24	182	67.8	Tennis	11
12	20	165	50.2	Tennis	8
13	21	166	65.3	softTennis	10
14	27	184	85.1	Baseball	15
15	23	171	73.5	Baseball	17
16	31	173	76.15	Baseball	23
17	25	180	76.15	Baseball	18
18	27	175	85.1	Baseball	6
19	26	181	85.2	Baseball	14
20	38	170	80.5	Tennis	22
21	27	160	57.9	Tennis	14

**Table 2:** Inclusion and exclusion in this study.

Category	No.	Description
Inclusion criteria	1.	Regular participation in an upper-extremity-dominant sport
	2.	5 years of uninterrupted practice in the sport
	3.	Subjective performance decline within the past 3 months
Exclusion criteria	1.	Presence of a diagnosed cardiovascular, psychiatric, or other serious medical condition use of a cardiac pacemaker or other implanted electronic medical device
	2.	Ongoing treatment or incomplete rehabilitation for any musculoskeletal or systemic injury or illness
	3.	Current pregnancy or the possibility of pregnancy
	4.	Atopic dermatitis or other dermatological conditions at the site of device attachment (forearm)
	5.	Clinically diagnosed anemia
	6.	General poor physical condition as assessed on the day of participation
	7.	Clinically diagnosed anemia
	8.	Presence of open wounds, skin irritation, or localized muscle soreness on the forearm
	9.	Insufficient sleep (less than 7 h) on the day of the experiment
	10.	Alcohol consumption on the day before or on the day of the experiment
	11.	Insect bites, scratches, or other localized skin damage at the sensor attachment site
	12.	Recent blood donation (within several days prior to the experiment)
	13.	Fasting or experiencing significant hunger at the time of participation
	14.	Occupation or regular activity involving repetitive or intensive use of the wrist (e.g., playing musical instruments)
	15.	Determination by the experimenter that the participant exhibits abnormal physical or psychological status that may interfere with data collection or safety

### 2.2 Equipment

#### 2.2.1 Experimental procedures

##### 2.2.1.1 Muscle deformation data acquisition

Muscle deformation data were compared by performing exercises with different loading amounts on the muscles, hand-push tasks, and movements with competition characteristics. The training with different amounts of the load was based on the report of Miyake et al. (21).

**Figure 1:**
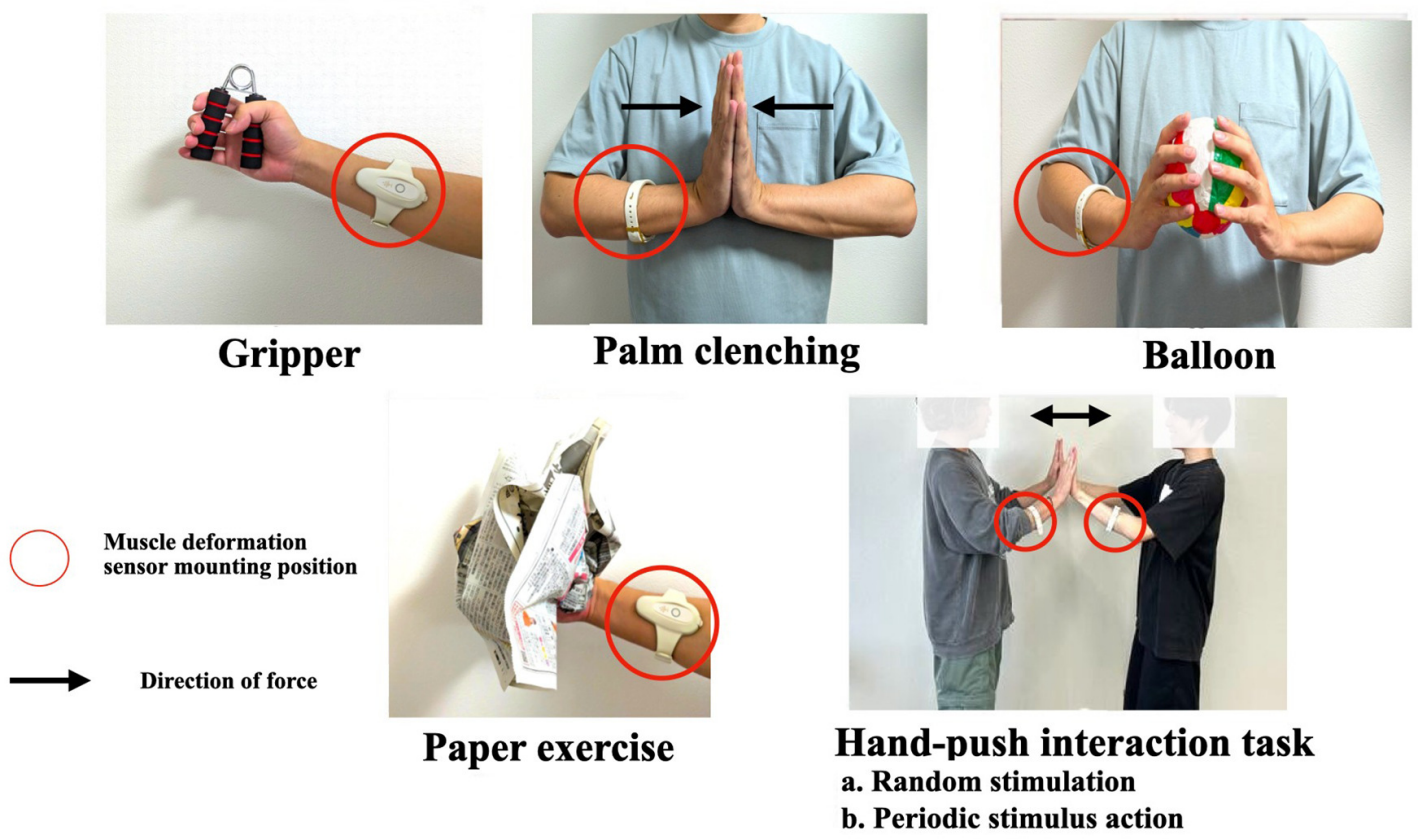
Performing exercises. The gripper and paper tasks were isotonic exercises, and the other tasks were isometric exercises. Hand-push interaction (HPI) was a task with periodic and random stimuli. In addition, crumpling pieces of paper exercise (paper) involves randomness because the resistance force of the paper changes depending on the shape and layers of the paper.

Tasks with different loading amounts as shown in Figure 1, participants performed the following five training tasks.

**Figure 2:**
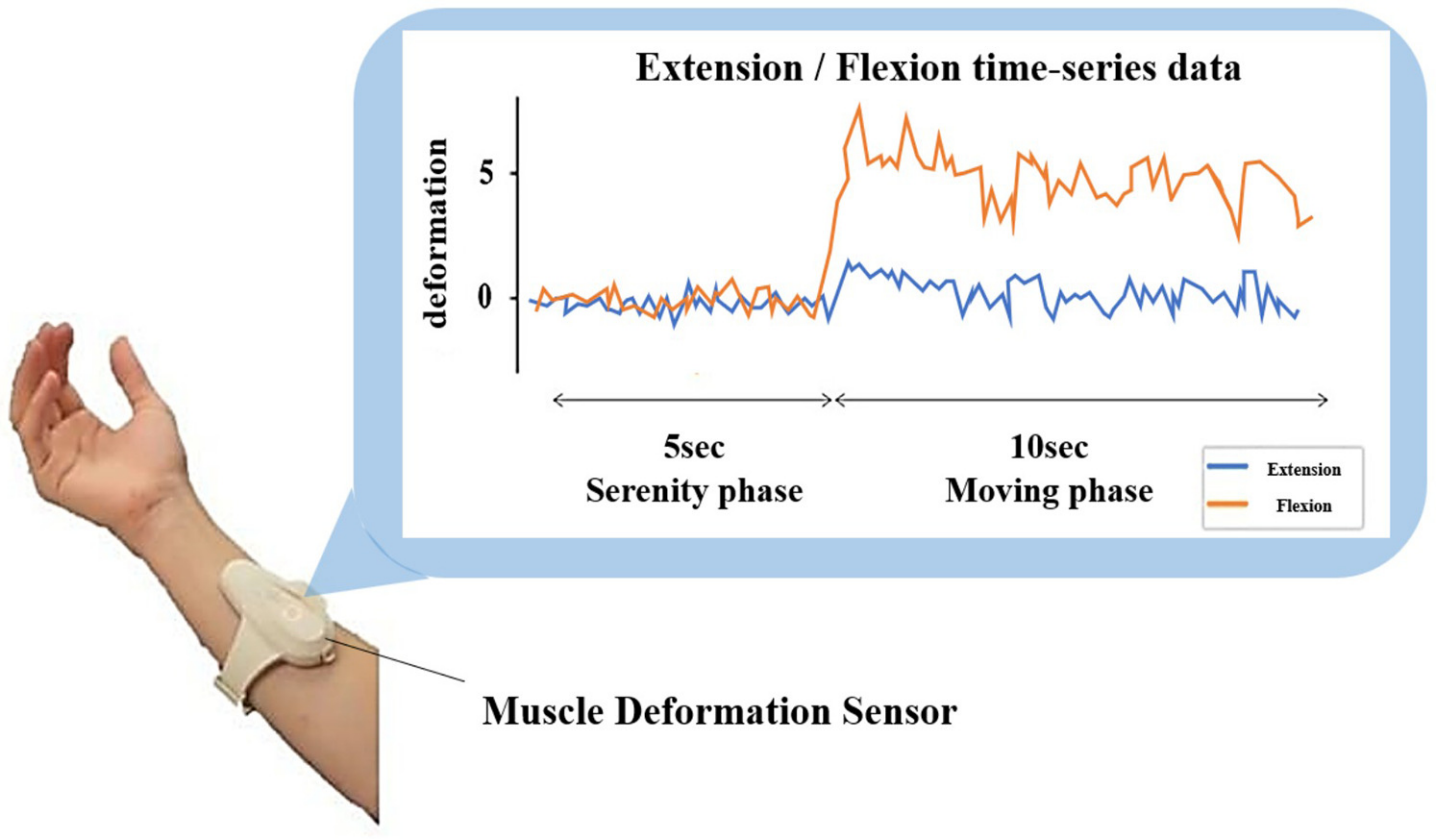
Summary of measurements and equipment used in the experiment. The entire training task was performed for 15 s. The first 5 s were used as the serenity phase, and the remaining 10 s were used as the moving phase. The moving phase consisted of grasping and other movements for 10 s. The muscle deformation sensor array used for the experimental task contained 14 optical sensors. When skeletal muscles are activated, the skeletal muscle deforms by muscle contraction. The reading value in each channel of the muscle deformation sensor changes when skeletal muscle or skin deforms. The reading values were sent to a computer from a muscle deformation sensor through Bluetooth Low Energy. The device is also equipped with a gyro sensor and a 3-axis acceleration sensor, making it possible to acquire quaternion data.

For the first 5 s, the forearm orientation was the same as that used in the task to reduce the effects of skin deformation. During calibration data acquisition, the wrist angle was set to the forearm at $0^{\circ }$ to relax the skeletal muscles. After the first 5 s had elapsed, a training movement, such as grasping, was initiated for 10 s (Figure 2). Calibration was performed for each assignment.

• Gripper: Grip and release a 25 kg hand-gripper approximately eight times for 10 s.
• Palm clenching (Hand): Keep pushing the hand in front of the chest for 10 s. Pressing with the entire palm.
• Balloon: Hold a paper balloon in front of the chest and apply pressure with arms for 10 s, making sure not to crush the balloon.
• Paper exercise (Paper): Asked to keep crumpling a layer of newspaper for 10 s.
  The gripper and paper tasks were isotonic exercises, and the other tasks were isometric exercises. The gripper and tasks were selected as the most typical hand-gripping training, where the human moves the skeletal muscles periodically. In addition, crumpling pieces of paper involves randomness because the resistance force of the paper changes depending on the shape and layers of the paper.
• Hand-push interaction task: The subject and the experimenter performed a hand-push interaction for 10 s with their hands clasped before their chests (Figure 1). The following two tasks were performed during the hand-push interaction:
  a. Random stimulation (HPI Randomness)
    The experimenter applied random perturbations to the subjects three times, and the subjects maintained their balance.
  b. Periodic stimulus action (HPI Periodicity)
    A metronome was played at 60 BPM, and the experimenter and the subject alternately pushed each other at 1 s intervals. This interaction was repeated ten times per trial.

##### 2.2.1.2 Psychological factor

Psychological conditions were first checked by questionnaire before the experiment. The questionnaires used the Kessler 6 scale(K6) (Table 3).

**Table 3:** K6 questionnaire.

During the past 30 days, how often did you experience anyof the following?
**Q1**. Did you feel irritable?
**Q2**. Did you feel hopeless?
**Q3**. Did you feel fidgety or restless?
**Q4**. Did you feel depressed and unable to get up to speed on what was happening?
**Q5**. Did you feel that everything you did was a struggle?
**Q6**. Did you feel that you are worthless?

Each item is scored from 0 to 4 (none to all of the time) and summed (range 0–24), with higher scores indicating greater distress.

The K6 is a brief 6-item self-report measure designed to assess non-specific psychological distress (such as anxiety and depression) experienced over the past 30 days (22). Higher scores indicate a greater risk of psychological distress, including anxiety and depression. The K6 was originally written in English, and a Japanese version has been prepared. The Japanese version was used in this study, (23). The K-6 has demonstrated very good internal consistency and excellent predictive validity (24). The K6 has also been utilized in studies involving athletes, including both general sports practitioners and elite athlete populations. Among these groups, the K6 has demonstrated high internal consistency (25–27). Furthermore, it has been employed as a criterion measure to validate athlete-specific psychological assessments, such as the Athlete Psychological Strain Questionnaire (APSQ), showing strong convergent validity (r = 0.80) (28).

##### 2.2.1.3 Equipment used in this study

This study used a muscle deformation sensor array, FirstVR (H2L Inc, Tokyo, Japan) (29), as shown in Figure 2. The FirstVR is a band-type sensor based on near-infrared optical sensing, which consists of infra-light emitting and infra-light receiving. The muscle deformation sensor array contains 14 optical muscle deformation sensors. When skeletal muscles are activated, the skeletal muscle deforms by muscle contraction. The reading value in each channel of the FirstVR changes when the skeletal muscle or skin deforms. The attachment cover was modified by replacing the Urethane sponge from the original FirstVR to observe muscle deformation. The reading values were sent to a computer from FirstVR through Bluetooth Low Energy. The sampling frequency of the data recording was approximately 9 Hz. The sensor is mounted approximately one-half of the forearm length from the elbow. The device is also equipped with a gyro sensor and a 3-axis acceleration sensor, making it possible to acquire quaternion data (posture data of the part of the body wearing the device). The device can be worn by wrapping it around the forearm like a wristwatch to measure muscle deformation in the forearm and fingers (30).

##### 2.2.1.4 Data processing and analysis

This study subjected the muscle deformation data obtained from the muscle deformation sensor array to moving average processing. The forearm muscle deformation data were categorized as follows. The forearm was defined as (1) Extensor muscles and (2) Flexor muscles. The observed flexor muscles are flexor carpi ulnaris muscle and flexor carpi radialis muscle. The observed extensor muscles are extensor carpi ulnaris muscle and extensor carpi radialis muscle. Autocorrelation coefficients were obtained based on muscle deformation time-series data obtained from each task. In this study, autocorrelation was used to evaluate the temporal regularity of muscle deformation patterns in response to task structure. This approach is justified by the fact that muscle activation during motor tasks often reflects rhythmic coordination when the external input is temporally structured. Conversely, random task conditions tend to disrupt this regularity. Autocorrelation thus serves as a physiologically relevant metric to assess how well muscle responses temporally align with the external task dynamics. The autocorrelation coefficient is the correlation between the original and time-shifted data. The autocorrelation coefficient is high if the time-series data are periodic (31). If the autocorrelation coefficient is low, it indicates that the data is random. This study used extreme values of the autocorrelation coefficient (except for the zero phase difference). Psychological distress levels were represented as a dummy variable based on the K6 score results. Statistical analysis examined the relationship between the autocorrelation coefficients of muscle deformation in each task and psychological distress measured by the K6. Statistical analysis was conducted with the effect size set at 0.6, the power of the test at 0.8, and a significance level of 0.05. Based on these parameters and the correlation test setup, the required sample size was calculated as 18 participants using the R pwr package. The first analysis was performed using the Shapiro-Wilk test to confirm the normality of the compared data. Subsequently, Pearson’s product-moment correlation coefficient or Spearman’s rank correlation coefficient was calculated depending on the distribution of the data. Furthermore, FDR correction using the Benjamini–Hochberg method was applied to account for multiple comparisons. Finally, additional group comparisons were made by dividing participants into two groups: the top five K6 scorers (four with six points and one with nine points) and the bottom five scorers (all with zero points). Group differences were assessed using the Wilcoxon rank-sum test.

##### 2.2.1.5 Ethical considerations

The experiment was conducted in accordance with the Declaration of Helsinki. All participants were given a written and verbal explanation of the experiment. All experimental tasks were performed with the consent of the participants. They were also explained so that participants could discontinue the experiment or withdraw their consent at any time. This experiment was approved by the Japanese Society for Assistive Technology (No. 23-05).

## 3 Results

This study analyzed the relationship between the autocorrelation of muscle deformation and psychological factor in each task.

**Figure 3:**
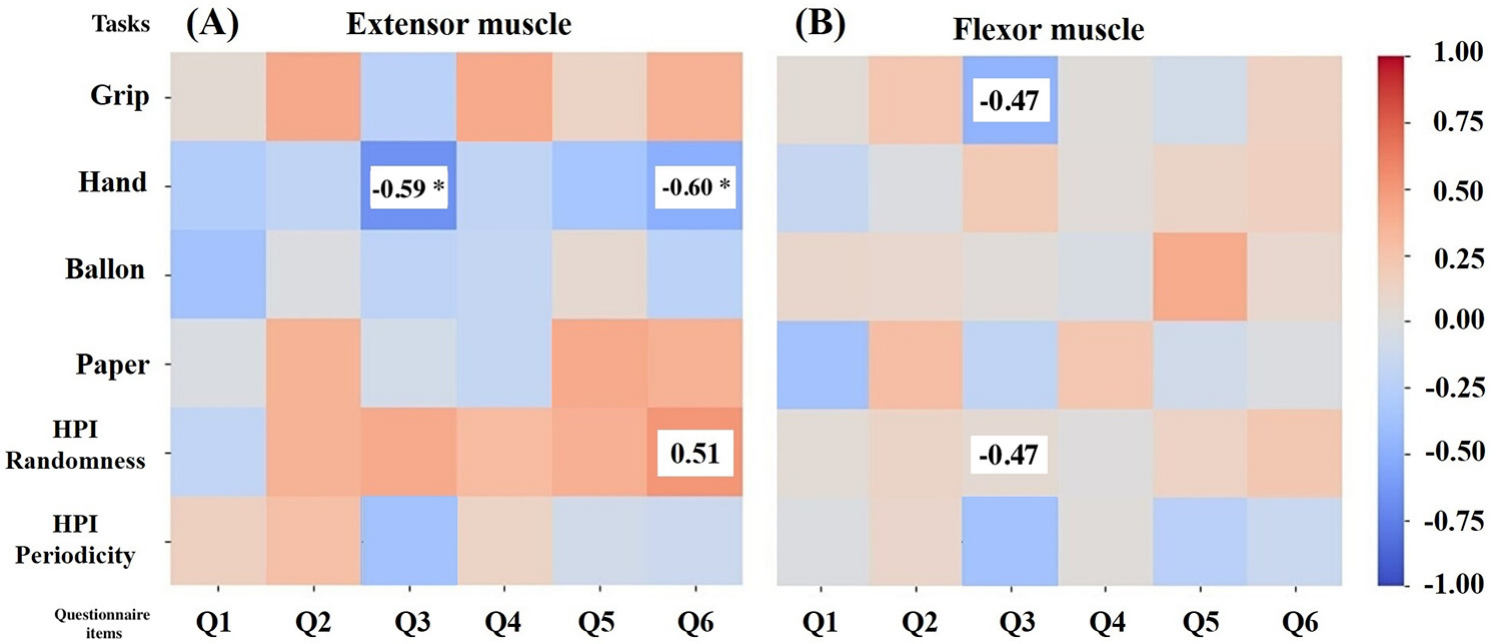
Correlation between Autocorrelation Coefficient of Muscle Deformation and K6 for Each Training. Heat-map of Spearman correlations between psychological items and muscle indices. (**A**) Extensor group; (**B**) Flexor group. The vertical axis of the figure shows the gripper, Hand, balloon, paper, HPI randomness and HPI periodicity tasks. The horizontal axis of the figure represents Q1–Q3 = anxiety items, Q4–Q6 = depression items. The items that showed predominant correlations were: (**A**) Q3 and Hand (r = −0.59), Q6 and Hand (r = −0.60), and HPI Randomness task (r = −0.51). (**B**) For flexors, Q3 and gripper (r = −0.47) and HPI Randomness task (r = −0.47). The heat map shows a positive correlation with a maximum of 1 in red and a negative correlation with a maximum of −1 in blue. Values shown in the heat map represent results after *p*-value correction. Values with an asterisk (*) represent p<0.05, values without an asterisk (*) represent p<0.10.

To examine how anxiety and depression are related to the autocorrelation coefficient of muscle deformations, the K6 scale was used. The results for K6 were: total score 3.2 ± 2.6, Anxiety score (Q1–Q3) 1.8 ± 1.4, Depression score (Q4–Q6) 1.5 ± 1.5, Q1: 0.6 ± 0.8, Q2: 0.5 ± 0.5, Q3: 0.7 ± 0.9, Q4: 0.5 ± 0.7, Q5: 0.6 ± 0.6, Q6: 0.4 ± 0.7. The correlations between each task and the total K6 scores, anxiety item scores, depression item scores, and each question are shown in Tables 4, 5. K6 total scores showed a significant negative correlation with forearm extensor deformity in the Hand task (r = −0.57). Muscle deformation of the forearm extensor muscles correlated with the Hand task (r = −0.58) and the HPI randomness task (r = 0.46) in anxiety (Q1–Q3) and depression (Q4–Q6). Additionally, Table 5 and Figure 3 suggest associations between each K6 problem and specific tasks. Extensors in Figure 3A were significantly correlated with Q3 (r = −0.59) and the Hand, as well as with Q6 and the Handr = −0.60), HPI randomness task (r = −0.51). For the flexor muscles in Figure 3B, associations were found between Q3 and the gripper task (r = −0.47) and the HPI randomness task (r = −0.47). Finally, the five highest and lowest K6 scores were compared between the high- and low-stress groups. No statistically significant differences were found between the groups. However, moderate to large effect sizes were observed in extensor Gripper (r = 0.30), Hand (r = 0.60), balloon (r = 0.49) and HPI randomness (r = 0.54) and flexor balloon (r = 0.49) and HPI periodicity (r = 0.30) (Table 6).

**Table 4:** Autocorrelation coefficient of muscle deformity in relation to K6 and total scores on anxiety and depression items for Each Training.

Score	Task	Extensor					Flexor				
		r	p	$p_{\mathrm{fdr}}$	CI		r	p	$p_{\mathrm{fdr}}$	CI	
Total score	Gripper	0.20	0.38	0.71	−0.25	0.58	−0.14	0.56	0.78	−0.54	0.31
	Hand	−0.57	0.01*	0.05	−0.80	−0.17	0.07	0.78	0.78	−0.38	0.48
	Ballon	−0.09	0.71	0.71	−0.50	0.36	0.16	0.49	0.78	−0.29	0.55
	Paper	0.10	0.67	0.71	−0.35	0.51	−0.12	0.61	0.78	−0.52	0.33
	HPI -R	0.39	0.08	0.24	−0.05	0.70	0.10	0.67	0.78	−0.35	0.51
	HPI- P	−0.13	0.57	0.71	−0.53	0.32	−0.28	0.22	0.78	−0.63	0.17
Anxiety score (Q1–3)	Gripper	0.02	0.94	0.94	−0.42	0.45	−0.17	0.45	0.87	−0.56	0.28
	Hand	−0.58	0.01*	0.06	−0.81	−0.20	0.04	0.87	0.87	−0.40	0.46
	Ballon	−0.20	0.39	0.78	−0.58	0.25	0.07	0.75	0.87	−0.37	0.49
	Paper	0.05	0.84	0.94	−0.39	0.47	−0.20	0.38	0.87	−0.58	0.25
	HPI -R	0.29	0.21	0.63	−0.17	0.64	0.08	0.74	0.87	−0.37	0.49
	HPI -P	−0.12	0.61	0.92	−0.52	0.33	−0.30	0.19	0.87	−0.65	0.15
Depression score (Q4–6)	Gripper	0.39	0.08	0.16	−0.05	0.70	−0.02	0.94	0.97	−0.45	0.42
	Hand	−0.44	0.05	0.15	−0.73	−0.01	0.08	0.73	0.97	−0.36	0.49
	Ballon	0.03	0.89	0.89	−0.40	0.46	0.24	0.30	0.97	−0.22	0.61
	Paper	0.18	0.43	0.65	−0.27	0.57	0.01	0.97	0.97	−0.42	0.44
	HPI -R	0.46	0.04*	0.15	0.03	0.74	0.10	0.68	0.97	−0.35	0.51
	HPI -P	−0.08	0.72	0.86	−0.50	0.36	−0.13	0.57	0.97	−0.53	0.32

p, *p*-value; $p_{\mathrm{fdr}}$, adjusted *p*-value (FDR); CI, confidence interval; HPI-R, HPI randomness; HPI-P, HPI periodicity; *significant difference.

Tasks related to anxiety (Q1–Q3) and depression (Q4–Q6).

The effect size is specified as 0.1 for small, 0.3 for medium, and 0.5 for large.

**Table 5:** Correlation between autocorrelation coefficient of muscle deformation and K6 for each training.

Question	Task	Extensor					Flexor				
		r	p	$p_{\mathrm{fdr}}$	CI		r	p	$p_{\mathrm{fdr}}$	CI	
Q1	Gripper	0.04	0.87	0.87	−0.40	0.46	0.04	0.85	0.93	−0.39	0.47
	Hand	−0.26	0.26	0.78	−0.62	0.19	−0.16	0.48	0.93	−0.56	0.29
	Ballon	−0.23	0.32	0.78	−0.60	0.22	0.09	0.68	0.93	−0.35	0.51
	Paper	−0.08	0.72	0.86	−0.50	0.36	−0.37	0.10	0.60	−0.69	0.08
	HPI -R	−0.18	0.45	0.78	−0.30	0.55	−0.02	0.93	0.93	−0.45	0.41
	HPI -P	0.15	0.52	0.78	−0.56	0.28	0.04	0.88	0.93	−0.40	0.46
Q2	Gripper	0.42	0.06	0.32	−0.02	0.72	0.23	0.32	0.88	−0.23	0.60
	Hand	−0.18	0.43	0.52	−0.57	0.27	−0.02	0.92	0.92	−0.45	0.41
	Ballon	0.15	0.52	0.52	−0.30	0.55	0.08	0.73	0.88	−0.37	0.49
	Paper	0.32	0.16	0.32	−0.13	0.66	0.29	0.20	0.88	−0.16	0.64
	HPI -R	0.36	0.11	0.32	−0.18	0.63	0.10	0.66	0.88	−0.34	0.51
	HPI -P	0.28	0.23	0.35	−0.08	0.69	0.11	0.63	0.88	−0.34	0.52
Q3	Gripper	−0.21	0.37	0.48	−0.59	0.25	−0.47	0.03*	0.09	−0.75	−0.05
	Hand	−0.59	0.004*	0.02*	−0.82	−0.22	0.20	0.39	0.62	−0.25	0.58
	Ballon	−0.19	0.40	0.48	−0.58	0.26	0.03	0.90	0.90	−0.41	0.45
	Paper	−0.01	0.96	0.96	−0.44	0.42	−0.19	0.41	0.62	−0.58	0.26
	HPI -R	0.41	0.06	0.12	−0.73	0.00	−0.47	0.03*	0.09	−0.75	−0.05
	HPI -P	−0.43	0.05	0.12	−0.02	0.72	0.06	0.79	0.90	−0.38	0.48
Q4	Gripper	0.50	0.02*	0.12	0.09	0.77	0.02	0.94	0.99	−0.42	0.45
	Hand	−0.09	0.69	0.71	−0.50	0.35	0.03	0.90	0.99	−0.41	0.45
	Ballon	−0.16	0.48	0.71	−0.56	0.29	−0.04	0.87	0.99	−0.46	0.40
	Paper	−0.09	0.71	0.71	−0.50	0.36	0.23	0.32	0.99	−0.23	0.60
	HPI -R	0.31	0.17	0.51	−0.33	0.53	0.02	0.93	0.99	−0.42	0.45
	HPI -P	0.12	0.60	0.71	−0.14	0.65	0.00	0.99	0.99	−0.43	0.43
Q5	Gripper	0.10	0.67	0.69	−0.35	0.51	−0.08	0.73	0.73	−0.49	0.36
	Hand	−0.32	0.16	0.32	−0.66	0.13	0.11	0.62	0.73	−0.33	0.52
	Ballon	0.24	0.29	0.44	−0.21	0.61	0.40	0.07	0.42	−0.04	0.71
	Paper	0.44	0.04*	0.24	0.01	0.73	−0.09	0.70	0.73	−0.50	0.36
	HPI -R	0.38	0.09	0.27	−0.50	0.35	−0.24	0.29	0.73	−0.61	0.21
	HPI -P	−0.09	0.69	0.69	−0.06	0.70	0.13	0.57	0.73	−0.32	0.53
Q6	Gripper	0.34	0.13	0.26	−0.11	0.67	0.14	0.54	0.81	−0.31	0.54
	Hand	−0.60	0.004*	0.02*	−0.82	−0.22	0.16	0.49	0.81	−0.29	0.55
	Ballon	−0.01	0.95	0.95	−0.44	0.42	0.08	0.71	0.85	−0.36	0.50
	Paper	0.23	0.32	0.48	−0.22	0.60	−0.02	0.95	0.95	−0.44	0.42
	HPI -R	0.51	0.02*	0.06	−0.53	0.32	−0.14	0.53	0.81	−0.54	0.31
	HPI- P	−0.13	0.59	0.71	0.10	0.77	0.22	0.53	0.81	−0.23	0.60

p, *p*-value; $p_{\mathrm{fdr}}$, adjusted *p*-value (FDR); CI, confidence interval; HPI-R, HPI randomness; HPI-P, HPI periodicity; * significant difference.

The effect size is specified as 0.1 for small, 0.3 for medium, and 0.5 for large.

**Table 6:** Comparison of muscle activity metrics between high and low psychological distress groups (based on K6 scores).

Muscle group	Task	Hight	Low	p	Effect size	CI	
		(*n* = 5)	(*n* = 5)				
Extensor	Gripper	0.58 ± 0.06	0.45 ± 0.17	0.35	0.30	−0.11	0.35
	Hand	0.16 ± 0.04	0.33 ± 0.15	0.06	0.60	−0.39	0.01
	Ballon	0.26 ± 0.14	0.25 ± 0.07	0.75	0.49	0.69	0.85
	Paper	0.25 ± 0.11	0.23 ± 0.03	0.92	0.03	−0.10	0.20
	HPI -R	0.30 ± 0.09	0.17 ± 0.05	0.09	0.54	−0.04	0.23
	HPI -P	0.52 ± 0.11	0.55 ± 0.08	0.92	0.03	−0.19	0.12
Flexor	Gripper	0.52 ± 0.19	0.56 ± 0.19	1.00	0.00	−0.41	0.32
	Hand	0.27 ± 0.15	0.18 ± 0.05	0.68	0.13	−0.11	0.35
	Ballon	0.18 ± 0.06	0.19 ± 0.11	0.14	0.49	0.69	0.85
	Paper	0.35 ± 0.10	0.31 ± 0.09	1.00	0.00	−0.11	0.19
	HPI- R	0.32 ± 0.23	0.22 ± 0.08	0.53	0.20	−0.13	0.47
	HPI -P	0.35 ± 0.09	0.42 ± 0.10	0.35	0.30	−0.24	0.08

p, *p*-value; CI, confidence interval; HPI-R, HPI randomness; HPI-P, HPI periodicity.

High, a group of the top 5 K6 total scores (4 with 6 points and 1 with 9 points).

Low, a group of the bottom 5 K6 total scores (all 5 with 0 points).

The effect size is specified as 0.1 for small, 0.3 for medium, and 0.5 for large.

## 4 Discussion

This study examined the relationship between forearm muscle deformation and psychological distress (anxiety and depression) during various training tasks. The following sections discuss how long-term psychological symptoms may influence muscle activity patterns.

The relationship between psychological stress and athletic performance has been widely studied, particularly in the context of performance failures such as the yips. Previous studies have reported elevated levels of stress and social anxiety in affected athletes, with the yips conceptualized along a continuum between anxiety-related choking and task-specific dystonia (32, 33). These findings are primarily based on psychological assessments rather than physiological measurements or muscle-specific data. In contrast, the present study focuses on the physiological correlates of psychological distress during motor task execution. Prior research has shown that pressure-induced motor disturbances, such as choking or the yips, are often associated with abnormal neuromuscular activity, including increased forearm tension, co-contraction of antagonist muscles, and reduced movement variability or speed (34, 35). These maladaptive motor responses have been attributed to disruptions in sensorimotor integration or excessive conscious monitoring of automated motor patterns. By analyzing forearm muscle deformation patterns across structured tasks, this study offers a biomechanical perspective that complements existing psychological models of stress-related performance breakdowns. This approach may contribute to a more comprehensive understanding of how chronic psychological stress alters neuromuscular control in high-pressure environments.

In this study, anxiety and depression were assessed using the K6 questionnaire. Associations were observed between K6 scores and muscle deformation patterns during training tasks. Although these associations did not consistently reach statistical significance after multiple comparison corrections, they suggested a potential relationship between psychological distress and task-specific muscle activity. Negative trends were observed between K6 scores and extensor muscle deformation in the Hand and HPI Periodicity tasks, while positive trends appeared in the Gripper, Paper, and HPI Randomness tasks.

The K6 is a validated screening tool that assesses non-specific psychological distress, including anxiety and depression, over the past 30 days. These results suggest prolonged exposure to depressive symptoms may contribute to inconsistent muscle performance, particularly during tasks involving isometric contraction (e.g., Hand) and periodic movement (e.g., HPI Periodicity). Such patterns imply that chronic psychological stress may impair immediate motor output and interfere with the long-term adaptation and refinement of motor patterns during training. Although the results indicate possible associations, this study’s cross-sectional design limits causal interpretation. Therefore, these findings should be interpreted as correlational. The training tasks employed in this study included both open kinetic chain movements focusing on hand activity and closed kinetic chain modalities such as the HPI tasks (36). Motor execution can be functionally divided into focal and postural components. The focal component refers to movements that directly accomplish a task, whereas the postural component provides the necessary stabilization to support those movements (37). From a neurophysiological standpoint, these components are associated with distinct control pathways: the cortico-spinal tract, which governs fine motor control and dexterous finger movements (38), and the cortico-reticular-spinal tract (39), which primarily supports postural control (40). The former involves finger flexion and grasping, while the latter contributes to proximal stability, including shoulder and upper arm positioning. Effective use of forearm muscles depends on stable postural control, which requires predictive regulation by the central nervous system (41, 42). Based on the present findings, anxiety and depression appear to be associated with activity in both extensor and flexor forearm muscles during training. The cortico-reticular-spinal tract, which modulates extensor muscles, plays a key role in postural regulation. The observed association between extensor muscle deformation and psychological distress suggests that prolonged psychological stress may influence postural control. These changes may affect fine motor skills via cortico-spinal tracts and may also affect attentional control functions and have an impact on performance (43). Moreover, cerebellar mechanisms may also be involved in how anxiety and depression affect postural stability (44). Such involvement of central and peripheral pathways is consistent with previous reports showing that psychological stress can impair neuromuscular coordination by affecting timing, co-contraction, and movement stability and perception (45). These findings suggest that for individuals experiencing prolonged performance issues with comorbid anxiety and depression, incorporating elements of unpredictability and postural variability into training, rather than relying solely on repetitive, upper-extremity isometric exercises, may be beneficial for breaking maladaptive motor habits. Finally, the top and bottom five participants based on total K6 scores were classified into high- and low-stress groups for exploratory comparison. Although no statistically significant differences were found between groups, moderate to large effect sizes were observed in extensor tasks (Gripper, Hand, Balloon, HPI Randomness) and flexor tasks (Balloon, HPI Periodicity) (Table 6). The small sample size may have limited the statistical power of the analysis. Future studies should include a larger and more diverse sample, incorporating participants at different competitive levels to improve generalizability. Furthermore, as this study was cross-sectional, longitudinal research is needed to evaluate how anxiety and depression affect muscle performance and postural control over time.

## 5 Conclusions

This study was conducted with amateur athletes who complained of subjective discomfort. The physical state (changes in muscle status) during a training task was measured using a muscle deformation sensor array to determine the relationship between the autocorrelation coefficient of muscle deformation and anxiety and depression. The results showed that anxiety and depression showed a positive correlation in the randomness task and a negative correlation in the periodicity task. The correlation was mainly observed in the forearm extensor muscles. This study suggests that anxiety and depressive symptoms may hinder not only flexible muscle contraction responses but also continuous contraction and contraction-relaxation processes during random tasks. Therefore, understanding the psychological states of athletes, particularly those who subjectively perceive poor performance, is essential for interpreting muscle activity and the resulting physical performance. These findings provide valuable insights into performance impairments attributable to psychological factors. Importantly, our results support integrating anxiety-reducing strategies into athletic training programs. Interventions such as mental skills training, mindfulness-based techniques, relaxation strategies, or stress-targeted motor tasks may help improve neuromuscular coordination and motor adaptability under psychological stress. These approaches could enhance both mental resilience and physical performance, especially in athletes experiencing psychological burdens.

## Data Availability

The original contributions presented in the study are included in the article/Supplementary Material, further inquiries can be directed to the corresponding author/s.
